# Life events and hopelessness depression: The influence of affective experience

**DOI:** 10.1371/journal.pone.0187898

**Published:** 2017-11-27

**Authors:** Lihua Zhou, Jian Chen

**Affiliations:** 1 Educational Science College, Hengyang Normal University, Hengyang, Hunan province, China; 2 Development Research Center of Undergraduates, Hunan Institute of Technology, Hengyang, Hunan province, China; Xi'an Jiaotong University School of Medicine, CHINA

## Abstract

This study explored the association of the affective experience (AE) of life events on hopelessness depression (HD). Undergraduates (N = 301) participating in a 12-week prospective study completed measures of HD, cognitive style, and psychological stress. The results indicate AE is an underlying mechanism influencing the longitudinal link between life events and HD. Negative life events with clear negative AE directly promoted the development of HD. Positive life events with clear positive AE directly impeded the development of HD. Neutral life events with mixed AE directly and interacting with negative cognitive style promoted the development of HD. The results should increase understanding of the hopelessness theory of depression, and suggest that neutral life events should be important elements in depression therapy.

## Introduction

### Hopelessness theory and hopelessness depression

Based on learned helplessness theory of depression, researchers [1] proposed hopelessness theory of depression to illustrate some risk factors and the influence mechanism of these risk factors on hopelessness depression (HD). Negative life events (NLEs) and negative cognitive style are the major distal risk factors of the etiology chain of HD. NLEs directly set the etiology chain in motion, and combining with negative cognitive style increase the risk of hopelessness, which in turn, causes HD. The mechanism of these risk factors is called cognitive vulnerability-stress model of HD, which also is the core hypothesis of hopelessness theory. Hopelessness is a proximate and sufficient cause in the etiological chain. Researchers defined the symptoms caused by hopelessness as HD, which includes retarded initiation of voluntary responses, sad affect, negative cognitive style, suicide attempts and suicide ideation, lack of energy, sleep disturbance, difficulty in concentration, low self-esteem [1]. There has been vast research tested the application of cognitive vulnerability-stress model of HD among diverse participants over the past nearly 30 years [2, 3]. Needles and Abramson believe that the etiological chain also applies to the recovery pathway of HD, and they proposed a recovery model of HD based on the cognitive vulnerability-stress model [4]. They hypothesize that positive life events (PLEs) alone or interacting with an enhancing cognitive style will lead to regaining hopefulness (a proximate and sufficient cause), which in turn, facilitates an individual’s recovery from HD. Hence, recovery model is logically consistent with the hopelessness theory of depression and cognitive vulnerability-stress model [4]. Studies on this recovery model explored whether PLEs alone, or in combination with an enhancing cognitive style, was therapeutic for depression [4–6]. Some recent studies investigated the simultaneous effects of PLEs and NLEs on the course of depressive symptoms [7–10]. These studies provide a more comprehensive understanding of HD, and indicated that researchers should pay more attention to the link between overall life events and depression, not just specific life events. On the whole, the cognitive vulnerability-stress model and the recovery model of hopelessness theory are good research paradigms to explore the link between life events and depression. The prior studies in this area used a variety instruments to measure depressive symptoms [2], and most of them have focused on general depression rather than specific HD, although these studies fall within the framework of the hopelessness theory of depression. Therefore, the present study used the term “depression” to conduct a literature review. The present study critically examined the application of cognitive vulnerability-stress model and recovery model of HD while using a longitudinal follow-up investigation among Chinese undergraduates. In addition, we aimed to respectively investigate the mechanism of these influence factors.

### Life events and its classification

Life events are social and environmental circumstances that require psychological and physiological adaptation by an organism over time [11]. Fairbank and Hough [12] originally explored the implication of life events classification on the cause or consequences of depression. Qualitative aspects of events reflecting individual perceptions and interpretations are hypothesized to be crucial [13]. As an important source of stress, most existing researchers classify life events as PLEs and NLEs in terms of an individual’s subjective experience [9, 14, 15]. Life events that bring desirable affective experiences and benefit an individual’s mental health and social adaption are defined as PLEs, and life events that bring undesired affective experiences and have deleterious effects on an individual’s mental health and social adaption are defined as NLEs. In emotion research field, most researches focus on NLEs, while little researches focus on PLEs. Human affective experience involves a mix of multiple and complicated affective components [16]. Some life events with mixed affective experience, which involve desirable experiences and undesirable experience are defined as neutral life events (NeuLEs) or mixed life events [17]. Some researchers proposed that NeuLEs’ emotions are far less intense than PLEs and NLEs. NeuLEs are associated with positive and negative emotions [18]. From this perspective, PLEs and NLEs contain clear desirable or undesirable affective experiences, whereas the affective experience of NeuLEs is mixed. Thus, separating NeuLEs from NLEs and PLEs could make the affective experiences of NLEs and PLEs clearer.

### Life event and hopelessness depression

NLEs and PLEs are neither necessary nor sufficient conditions for hopelessness depression, but they are “occasion setters” in the etiological chain and the recovery pathway, which are features of hopelessness theory. They are highly important contributors that can facilitate or impede the development of hopelessness depression. Previous studies have contributed to our understanding of the course of depression. A substantial number of prior studies demonstrated that NLEs are robust risk factors for depression [19] and PLEs are associated with reduced risk for depression or the remission of depressive symptoms [9]. Nevertheless, we still do not know much about the mechanism by which these life events influence HD. In addition, some studies have reported inconsistent results regarding the model of cognitive vulnerability-stress and recovery model of HD. For example, the results of a recent study of undergraduates [9] showed that cognitive vulnerability interacting with NLEs predicts increased depression over time, which is consist with the cognitive vulnerability-stress model of HD. Wu et al reported that stress life events can predicted the change of depression and negative cognitive style has no significant moderate effect on the relationship between NLEs and depression among Chinese child [20]. One study reported that enhancing cognitive style and PLEs influence the relief of depression, independently, rather than in combination [14]. This study used the Acute Life Events Questionnaire to measure NLEs and PLEs. An earlier prospective study found that positive attributional style had mediating and moderating effects on the relationship between PLEs and depressive symptoms among children (10–12 years old) [15]. This study measured NLEs with the Cambridge Life Development Measure [21] and it measured PLEs with the Positive Life Events Checklist (which was developed by the authors). These studies all classified life events as PLEs and NLEs. A person’s categorization of life events is the outcome of cognitive evaluation in term of the weight of the negative and positive affective experiences [17]. If we only divide life events into NLEs and PLEs, then NeuLE may mix with PLEs or NLEs, which confuses the affective experience of these events. In other words, doing so can make the affective experience of life events mixed and produce more complex relationships among these events, cognitive style, and depression symptoms. Inconsistent results of studies on cognitive vulnerability-stress among children [20, 22, 23] can be explained partly by this inference. Some researchers believe that children may adopt a more neutral cognitive style [9]. From this point of view, children may consider most life events as NeuLEs. Therefore, we postulate a possible explanation that the mixed affective experience of NeuLEs may contribute to the discrepancies in the results in some prior studies.

### The role of cognitive style

Cognitive style in the hopelessness theory of depression affects inferences about the causes and consequences of life events and their implications for the self. Negative cognitive style is defined as making negative attributions or reasoning about causes, one’s self-worth, and the future consequences of NLEs [1]. Enhancing cognitive style is defined as making positive attributions or reasoning about causes, one’s self-worth, and the future consequences of PLEs [4]. The risk of suffering from depression increases when individuals with a negative cognitive style experience NLEs [1]. The possibility of remission of or recovery from depression increases when individuals with an enhancing cognitive style experience PLEs [4]. Prior studies demonstrate that negative interpretation of ambiguous information plays an important role in the development of depression [24]. Negative cognitive bias increases the frequency and intensity of negative emotion, which in turn, affects depressive symptoms [25]. Ambiguous information with respect to life events implies a mixed affective experience. So, based on research on cognitive biases and hopelessness depression, we deduced that affective experience is an underlying mechanism that influences the longitudinal link between life events and depression. If NeuLEs are separated from NLEs and PLEs, this will make the affective experience of NLEs and PLEs clearer, and NLEs and PLEs will affect the development of depression more directly. Individuals who have NeuLEs (which involve mixed affective experience) and focus on the negative components of the NeuLEs (individuals with negative cognitive style) will have a higher risk of depression according to the cognitive vulnerability-stress model of HD. On the other hand, individuals who focus on the positive components of NeuLEs (individuals with an enhancing cognitive style) will have a greater possibility of remission of the depression, according to the recovery model of HD. In other words, cognitive style will have a moderating effect on the relationship between NeuLEs and depression because of the ambiguous affective experience of NeuLEs.

### Overview of the current study

**Fig 1:**
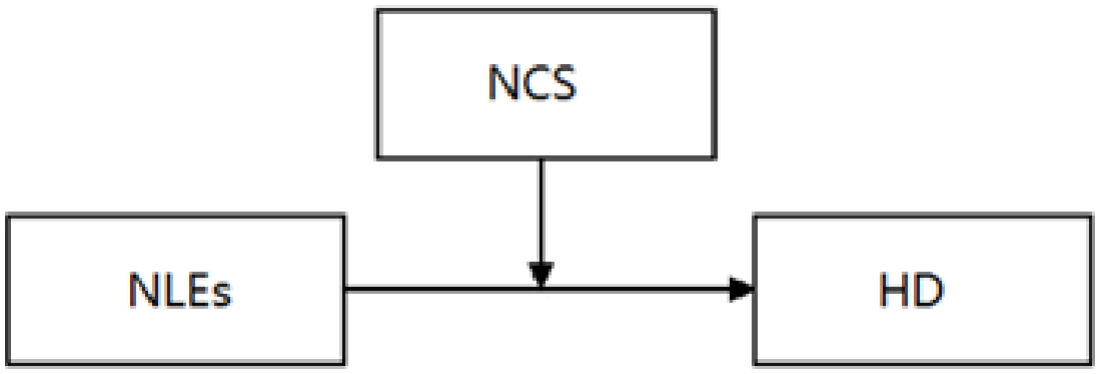
Effects of negative life events and negative cognitive style on hopelessness depression. Note: NLEs = Negative Life Events; NCS = Negative Cognitive Style; HD = Hopelessness Depression.

**Fig 2:**
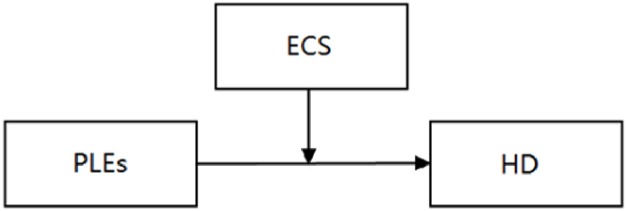
Effects of positive life events and enhancing cognitive style on hopelessness depression. Note: PLEs = Positive Life Events; ECS = Enhancing Cognitive Style; HD = Hopelessness Depression.

**Fig 3:**
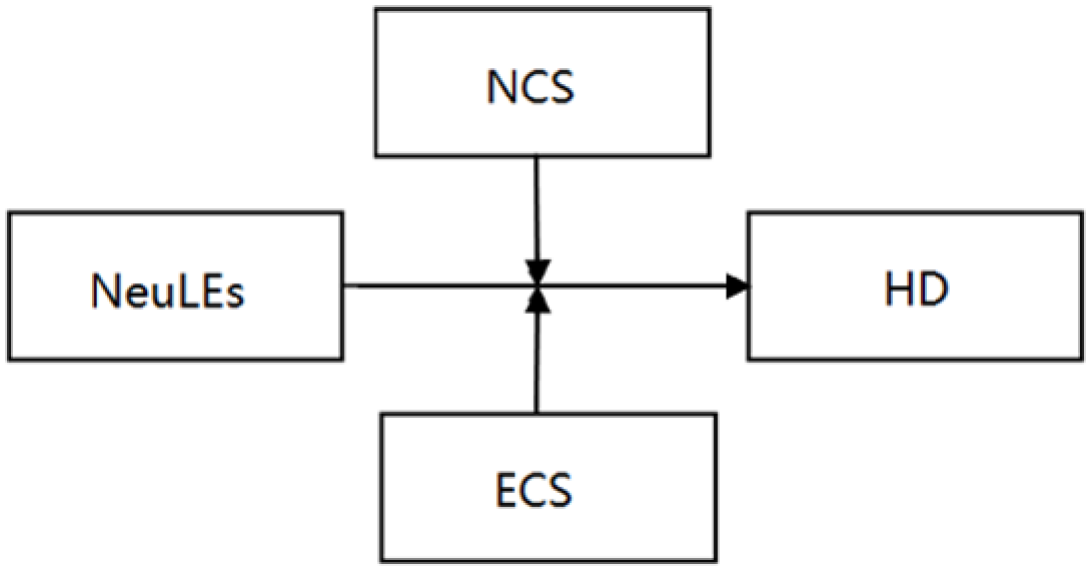
Effects of neutral life events and cognitive style on hopelessness depression. Note: NeuLEs = Neutral Life Events; NCS = Negative Cognitive Style; ECS = Enhancing Cognitive Style; HD = Hopelessness Depression.

The goal of the current study was to explore whether affective experience serves as an underlying mechanism that influences the longitudinal link between life events and depression. The present study analyzed the direct effects of different life events and the moderating effects of cognitive style on the association of different life events and HD symptoms within the hopelessness theory framework. The concept of the present study was depicted in Figs 1–3.

Based on our literature review, the current study hypothesized these possible relationships among life events, cognitive style and HD:

Hypothesis 1: NLEs with a clear undesirable affective experience will promote the development of HD symptoms directly.
Hypothesis 2: PLEs with a clear desirable affective experience will impede the development of HD symptoms directly.
Hypothesis 3: NeuLEs with some undesirable affective experience will increase HD symptoms in combination with negative cognitive style.
Hypothesis 4: NeuLEs with some desirable affective experience will decrease HD symptoms in combination with enhancing cognitive style.

To achieve this goal, the present study simultaneously analyzed these relationships: (1) NLEs and negative cognitive style as predictors of HD (cognitive vulnerability-stress model of HD); (2) PLEs and enhancing cognitive style as predictors of HD (recovery model of HD); (3) NeuLEs and negative cognitive style as predictors of HD; and (4) NeuLEs and enhancing cognitive style as predictors of HD. Identifying which of these hypotheses is correct should improve our understanding of the etiology and recovery course of HD and have important implications for designing preventive interventions.

## Methods

### Participants

Approval for the study was given by the Scientific Research Office of Hengyang Normal University. Participants were recruited in mental health education class and counseling psychology class. Three hundreds and eighteen undergraduates from Hengyang Normal University in Hunan province of China who volunteered for the study, 301 volunteers properly completed all measurement in the first time. The sample included 115 males (mean age = 19.46 years, *SD* = 0.88) and 186 females (mean age = 20.12 years, *SD* = 1.13). Among the 301 volunteers, 5 males and 8 females were in psychotherapy in school and 1 female was taking anti-psychotic medication. We kept these subjects in sample, because social functions of these subjects have not been seriously damaged, and they still could participate in school routine activities normally.

### Measures

#### Hopelessness depression symptom questionnaire (HDSQ)[26]

The HDSQ, which has 32 items, 8 subscales, was used to assess the severity of HD experienced in the past 2 weeks. The total scores of the HDSQ ranges from 0 to 96, with a higher score indicating more serious HD symptoms. The HDSQ has been demonstrated to have good reliability and validity. The Chinese version of the HDSQ was translated and validated by Yi and Feng [27].

#### Cognitive style questionnaire (CSQ)[2]

The CSQ was used to assess participants’ inferences about 12 hypothetical NLEs and 12 hypothetical PLEs. Participants were asked to write down the major causes of these events as if these events were happening in real time. Then, they used a 7-point Likert-type scale to rate the causes and consequences of these events and their implications for self-worth. An individual’s negative cognitive style score was the average rating for the 12 hypothetical NLEs. Similarly, an individual’s enhancing cognitive style score was the average rating for the 12 hypothetical PLEs. The composite score (total score divided by the number of items) ranges from 1 to 7, with higher scores indicating more negative or enhancing cognitive styles. The CSQ has satisfactory internal consistency, reliability, and validity [2]. The Chinese version of HDSQ was translated and validated by Chen, Yan, Zhou, and Su [28].

#### Chinese college student psychological stress scale (CCSPSS)[17]

The 85-item CCSPSS was used to assess life events and their psychological influence index (PII); each item represents one life event. The PII reflects the level of stress or psychological pressure caused by life events. The measure involves multiple aspects of life, including learning, daily living, society, development, and family. Participants are instructed to classify life events as PLEs, NLEs, or NeuLEs by their affective experience. Thus, the CCSPSS contains three subscales (a PLEs subscale, a NLEs subscale, and a NeuLEs subscale); the scores on these subscales vary for different individuals. In order to establish each event’s normal PII, more than 50,000 undergraduates at 182 universities in China participated in a survey. The scale has good reliability and validity [17].

### Procedure

The present study used a 12-week prospective longitudinal design. All participants were administered an informed consent form and a brief demographics questionnaire at the start of the study. Participants were assessed at three time points separated by approximately 4 weeks. At Time 1, 301 participants properly completed measures of hopelessness depression symptoms (HDSQ), cognitive style (CSQ), and life events (CCSPSS) that had occurred during the last 4 weeks. We examined data from 301 participants who provided complete baseline data at Time 1. Rates of participation in the study vary slightly at each measurement time. At Time 2, 255 participants properly completed measures of life events that had taken place over the preceding 4 weeks (events that had occurred since T1) and HD symptoms. At Time 3, 258 participants properly completed measures of life events that had occurred during the preceding 4 weeks (events that had occurred since T2) and HD symptoms. Cognitive style is considered to have great degree of stability across the different ways of examining over time among adolescents [29], and the stability become increasingly stronger with increasing age [30, 31]. The present study only measured cognitive style in time1. In addition, we used 4 weeks as an interval based on past researches. Such as, Haeffel and Vargas [9] used a 4-week prospective longitudinal design to test the relationship among cognitive style, life events and depression in an undergraduates sample (n = 128). Some researchers considered that shorter time-frames provide more accurate, less biased recall of symptoms [32]. Therefore, four weeks as an interval was appropriate for tracking the symptoms of HD in the present study.

### Data analytic strategy

According to these data of the current study, we used Hierarchical Linear Modeling (HLM) to test the hypotheses. HLM has two advantages for the analysis of these data: (1) it estimates within-subject (Level 1 data) and between-subject (Level 2 data) variation simultaneously; and (2) it accommodates missing data at Level 1, but not at Level 2. The statistical package used in the current study was HLM 6.08.

## Results

The means, standard deviations, and intercorrelations of the study variables are presented in Table 1.

**Table 1:** Means, standard deviations, and intercorrelations of study variables.

	1	2	3	4	5	6	7	8	9	10	11	12	13	14
1. T1 HD	—													
2. T2 HD	.764***													
3. T3 HD	.512***	.599***	—											
4. T1 NLEs	.240***	.193**	.212**	—										
5. T2 NLEs	.133	.249***	.154*	.405***	—									
6. T3 NLEs	.205**	.258***	.359***	.390***	.522***	—								
7. T1 PLEs	-.256***	-.171*	-.128*	-.065	-.118	.068	—							
8. T2 PLEs	-.215**	-.181*	-.150*	-.093	.007	.077	.455***	—						
9. T3 PLEs	-.128*	-.046	-.119	.002	.009	.068	.399***	.500***	—					
10. T1 NeuLEs	.170**	.125	.142*	.170**	.061	-.032	-.264***	-.126	-.108	—				
11. T2 NeuLEs	.188**	.199**	.121	.146*	.424***	.170*	-.262***	-.047	-.089	.451***	—			
12. T3 NeuLEs	.310***	.226**	.171**	.104	.236**	.235***	-.165**	-.093	-.181**	.286***	.446***	—		
13. NCS	.241***	.232**	.164**	.214***	.223**	.100	-.104	-.155	-.120	-.062	.071	.123*	—	
14. ECS	-.057	-.067	-.077	.114*	.086	.153*	.155**	.125	.051	-.194**	-.078	.069	.278***	—
*M*	18.82	14.48	13.63	884.83	350.69	291.03	625.08	632.88	562.59	956.61	624.73	523.86	4.31	5.22
*SD*	8.52	7.20	7.51	563.31	341.22	344.67	305.54	417.52	407.27	467.39	468.63	556.74	0.72	0.58

Note. T1 = Time 1; T2 = Time 2; T3 = Time 3; NLEs = Negative Life Event of CCSPSS; PLEs = Positive Life Event of CCSPSS; NeuLEs = Neutral Life Event of CCSPSS; NCS = Cognitive Style Questionnaire; ECS = Enhancing Cognitive Style Questionnaire.

*p≤.05.

**p ≤.01.

***p ≤.001.

The hopelessness theory predicts that the relationship of NLEs and HD is moderated by negative cognitive style. To test this hypothesis, we used the procedure suggested by Kwon and Laurenceau[33], resulting in the following Level 1 model:

$$HD_{\text{ij}}=\beta_{0j}+\beta_{1j}(T_{i})+\beta_{2j}(NLEs_{ij})+r_{ij}$$ (1)

where *β*_0*j*_ represented participant *j*’s predicted HD value when Time equaled zero, *β*_1*j*_ represented the linear effect of time across occasions(i.e., measured time points), *β*_2*j*_ represented the change in HD for participant *j* for a corresponding change in NLEs, *T*_*i*_ represents the value of time from 0 to -2, *NLEs*_*ij*_ was the number of reported negative life events for occasion *i* and participant j, and *r*_*ij*_ was the residual term associated with observation *i* for participant *j*. The Level 2 model represented a null model as:

$$\beta_{0j}=\gamma_{00}+u_{0j}$$ (2)

$$\beta_{1j}=\gamma_{10}+u_{1j}$$ (3)

$$\beta_{2j}=\gamma_{20}+u_{2j}$$ (4)

The results of this Level 1 model and the corresponding Level 2 null model indicated that NLEs was a significant predictor of HD, *γ*_20_ = 1.913, *t*(299) = 6.910, *p* = .000.

To examine the moderating effect of negative cognitive style, the following Level 2 model was estimated:

$$\beta_{0j}=\gamma_{00}+\gamma_{01}(NCS_{j})+u_{0j}$$ (5)

$$\beta_{1j}=\gamma_{10}+\gamma_{11}(NCS_{j})+u_{1j}$$ (6)

$$\beta_{2j}=\gamma_{20}+\gamma_{21}(NCS_{j})+u_{2j}$$ (7)

The effect of negative cognitive style on the relationship between NLEs and HD was not significant, *γ*_21_ = -0.366, *t*(298) = -1.032, *p* = 0.303. The effect of negative cognitive style on the relationship between occasion and HD was not significant, *γ*_11_ = 0.464, *t*(298) = 1.124, *p* = 0.263.

We used the above method to examine the recovery model of HD; i.e., the moderating effect of enhancing cognitive style on the association between PLEs and HD. PLEs emerged as a significant predictor, *γ*_20_ = -0.821, *t*(299) = -3.465, *p* = 0.001. The effect of enhancing cognitive style on the relationship between PLEs and HD was not significant, *γ*_21_ = -0.646, *t*(298) = -1.564, *p* = 0.119. The effect of enhancing cognitive style on the relationship between occasion and HD was not significant, *γ*_11_ = 0.048, *t*(298) = 0.125, *p* = 0.901.

We also used the same method to examine the moderating effect of negative cognitive style and enhancing cognitive style in the relationship between NeuLEs and HD. NeuLEs emerged as a significant predictor, *γ*_20_ = 1.017, *t*(299) = 3.388, *p* = 0.001. The effect of enhancing cognitive style on the relationship between NeuLEs and HD was not significant, *γ*_21_ = -0.438, *t*(298) = -0.871, *p* = 0.385. The effect of enhancing cognitive style on the relationship between occasion and HD was not significant, *γ*_11_ = 0.307, *t*(298) = 0.692, *p* = 0.489. The effect of negative cognitive style on the relationship between NeuLEs and HD was significant, *γ*_21_ = -0.882, *t*(298) = -2.243, *p* = 0.026. The effect of negative cognitive style on the relationship between occasion and HD was significant, *γ*_11_ = 0.917, *t*(298) = 2.320, *p* = 0.021. Results of fixed effects of these models are summarized in Table 2.

**Table 2:** Fixed effects.

	Coefficient	SE	*t*	*df*	*p*
NLEs, NCS, and HD					
*γ*_20_	1.913	0.277	6.910	299	0.000
*γ*_21_	-0.366	0.355	-1.032	298	0.303
*γ*_11_	0.464	0.413	1.124	298	0.263
PLEs, ECS, and HD					
*γ*_20_	-0.821	0.237	-3.465	299	0.001
*γ*_21_	-0.646	0.413	-1.564	298	0.119
*γ*_11_	0.048	0.388	0.125	298	0.901
NeuLEs, NCS, and HD					
*γ*_20_	1.017	0.300	3.388	299	0.001
*γ*_21_	-0.882	0.393	-2.243	298	0.026
*γ*_11_	0.917	0.396	2.320	298	0.021
NeuLEs, ECS, and HD					
*γ*_21_	-0.438	0.502	-0.871	298	0.385
*γ*_11_	0.307	0.444	0.692	298	0.489

Note. NLEs = Negative Life Events; PLEs = Positive Life Events; NeuLEs = Neutral Life Events; NCS = Negative Cognitive Style; ECS = Enhancing Cognitive Style; HD = Hopelessness Depression.

## Discussion

The present study was designed to explore the influence of the affective experience of life events for HD. The present study makes the affective experiences of life events clearer and distinctly different from each other by classifying life events as PLEs, NLEs, and NeuLEs. There were some novel findings in the present study. First, the study’s results supported hypothesis 1 and hypothesis 2. They showed that NLEs directly contributed to an increase in HD symptoms and PLEs directly contributed to a decrease in HD symptoms. The present study did not find a significant moderating effect of cognitive styles on the relationships between NLEs and HD or PLEs and HD. Several existing studies that found NLEs [34] and PLEs [9, 10, 35] were predictors of depression. Other studies found that negative cognitive style had a moderating effect on the relationship between NLEs and HD [12, 36–39] in samples of adolescents, college students, and older adults (ranging from 59 years to 97 years-of-age), which is consistent with the cognitive vulnerability-stress model of HD. The results of some studies also supported the recovery model of HD and indicated that enhancing cognitive style moderated the association between PLEs and depressive symptoms among non-clinical samples of children and adolescents [5, 15], as well as outpatients [40, 41] and inpatients [6]. The results of other studies were partially consistent with our results. For example, a study of young adults found significant cognitive vulnerability-stress affected male but not female participants, and that negative cognitive styles and NLEs were independent predictors of depressive symptoms among female participants [42]. Another study found that an enhancing cognitive style and high levels of PLEs can buffer against increased depressive symptoms, but the protective effects of the two variables were independent, rather than interacting with each other [9]. The separating of NeuLEs in the present study highlighted the affective experience of NLEs and PLEs; thus, the two kinds of life events showed main effects on the change of HD.

The results of the present study provide support for the hypothesis 3, but not hypothesis 4, in that the relationship between NeuLEs and HD was moderated by a negative cognitive style but not an enhancing cognitive style. Thus, these findings support the application of the cognitive vulnerability-stress model to the associations among NeuLEs, negative cognitive style, and HD. Previous research has found that approximately 30% of the people who experienced their first onset of depression and 60% of these people who suffered a recurrence of depression did not recently have severely stressful life events [11]. Based on the findings of the current study, these people may have experienced a high rate of NeuLEs. The results of the present study may also be related to the age of the participants, in that the participants of the present study were undergraduates, and individuals of this age are more likely to seek positive stimulation from the environment. Relative to PLEs, NeuLEs do not supply a positive stimulus that young people find to be sufficient, which might produce lower physiological and emotional arousal and dissatisfaction with life. Thus, young people are the less likely to gain positive benefits from NeuLEs.

It is worth mentioning that NeuLEs had a significant main effect on the development of HD in the present study. NeuLEs were a potent risk factor for HD in that individuals who experienced more NeuLEs had a greater level of HD symptoms. Our literature review did not find research supporting these results directly, but some results of previous studies were indirectly related to the results of our study. Some researchers propose that although ambiguous life events could not be classified as positive or negative, they shared a common feature in requiring significant adaptation to change [43]. Hopelessness theory hypothesizes that either the occurrence of NLEs or the nonoccurrence of PLEs could set the etiology chain in motion [1]. Research also has demonstrated that the absence of PLEs might be depressogenic[9]. The present study found that only PLEs had a buffering effect on the development of HD symptoms; NLEs and NeuLEs both were significant risk factors for HD symptoms. In a 148 adolescents sample research, research found that NLEs were significantly associated with poorer adjustment, PLEs were associated with better adjustment, and ambiguous events were related to adjustment much in the same way as NLEs [43]. But the authors did not describe the classification method of life events in detail. Based on these results, one may speculate that even though NeuLEs contain some desirable components, these positive components are insufficient to produce a positive affective experience.

According to the combined results of the present study, the influence of NeuLEs on depression is consistent with the effects of NLEs and inconsistent with the effects of PLEs. Negative cognitive style only had a moderating effect on the relationship between NeuLEs and HD symptoms. When NeuLEs were mixed with NLEs, cognitive style only had a moderating effect on the relationship between NeuLEs and HD. Some researchers have concluded that a study’s operational definition of cognitive vulnerability [44, 45], research design, and differences in participant characteristics [46–48], the combination of a sample’s age and gender [49] may lead to mixed results about the association of cognitive vulnerability-stress with HD. As mentioned above, the present research indicated that the mixing of NLEs and NeuLEs may also contribute to differences in the moderating effect of negative cognitive styles. In addition, researchers do not seem to have much confidence about the beneficial effects of PLEs on the remission of or recovery from depression. For example, some researchers have noted that PLEs lead to an increased risk of depression [50], and other researchers have proposed that PLEs buffer against the effect of NLEs on depression effectively only when the number of PLEs to NLEs reaches a ratio of 2.9:1 (also known as the Rosa Ratio) [51, 52]. The results of the present study provide a novel explanation for these inconsistencies; when NeuLEs are mixed with PLEs, they contaminate the direction of an enhancing cognitive style’s moderating effect on the association between PLEs and HD symptoms.

The present study has both strengths and weakness. First, the study’s results will increase understanding of the hopelessness theory of depression by systematically exploring the effects of different life events on HD. NeuLEs were absent from research on emotion for a long time because it seemed that NeuLEs were not important for an individual’s emotions. Some researchers have suggested that it is necessary to examine a wider range of cognitive and environmental factors to understand the risks of depression [9]. The present study found that a NeuLE was a potent risk factor for HD among undergraduate students because its affective experience was mixed. As negative cognitive style had a significant moderating effect on the relationship between NeuLEs and HD, the results of this study also demonstrated that the cognitive vulnerability-stress model of HD was applicable to NeuLEs. Second, the results of the present study indicate that psychological therapy for depression should pay attention to the affective experience of life events, especially cognitive therapy for depression. The results of the current study indicate that affective experience is an underlying mechanism influencing the longitudinal link between life events and depression. Furthermore, the mixed affective experience of NeuLEs should highlight the cognitive vulnerability-stress effect. Cognitive therapy should help individuals to clarify their subjective experiences of life events, especially NeuLEs. Third, the study classified life events by participants’ affective experience. This classification method took into account the individual’s evaluations and different responses to the same life event. As a Chinese proverb says, fish drinking water know for themselves whether it is cold or warm. For example, some participants in our study regarded failing an exam as a NLE, some participants regarded it as a PLE, and other participants thought it was a NeuLE. The results of this study are helpful for understanding some of the discrepancies reported in previous studies. On the whole, NeuLEs should be brought into the study of emotion. This is not only a methodological issue but also a conceptual issue.

There are several limitations of the present study that should be mentioned. First, the study participants were all undergraduates. There may be age differences in cognitions about NeuLEs between children, adolescents, and older persons. A NeuLE may mean provide peace and quiet for the mind of children and older persons, which in turn, benefits mental health. Further studies should expand the age range of participants and analyze age differences. Second, although the present study explored the implications of different life events for HD, we did not analyze the interaction between different life events and cognitive styles. Third, the study’s participants were a nonclinical sample and the instruments we used reflected the framework of HD theory. Although the present study contributes to our understanding of the theory of HD and the mechanisms by which life events affect HD, the findings may have limited application for a broader understanding of the risk factors and protective factors for general depression.

In conclusion, our results suggest that affective experience serves as an underlying mechanism that influences the longitudinal link between life events and depression. Clearer affective experience of life events is relate with more direct relationship between life events and hopelessness depression development; ambiguous affective experience of life events will highlight the moderate effect of the negative cognitive style between life events and HD. In particular, NLEs with clear undesirable affective experiences are directly related to increased symptoms of HD, and PLEs with clear desirable affective experiences are directly related to decreased symptoms of HD; NeuLEs with mixed affective experience contribute to increased symptoms of HD, and negative cognitive style moderates the relationship between HD and NeuLEs.

## Supporting information

S1 File Data 1. (SAV)

S2 File Data 2. (SAV)
